# Two-dimensional enrichment analysis for mining high-level imaging genetic associations

**DOI:** 10.1007/s40708-016-0052-4

**Published:** 2016-05-13

**Authors:** Xiaohui Yao, Jingwen Yan, Sungeun Kim, Kwangsik Nho, Shannon L. Risacher, Mark Inlow, Jason H. Moore, Andrew J. Saykin, Li Shen

**Affiliations:** 1 Radiology and Imaging Sciences, Indiana University School of Medicine, 355 West 16th Street Suite 4100, Indianapolis, IN USA; 2 School of Informatics and Computing, Indiana University Indianapolis, Indianapolis, IN USA; 3 Biomedical Informatics, School of Medicine, University of Pennsylvania, Philadelphia, PA USA

**Keywords:** Imaging genetics, Enrichment analysis, Genome-wide association study, Quantitative trait

## Abstract

Enrichment analysis has been widely applied in the genome-wide association studies, where gene sets corresponding to biological pathways are examined for significant associations with a phenotype to help increase statistical power and improve biological interpretation. In this work, we expand the scope of enrichment analysis into brain imaging genetics, an emerging field that studies how genetic variation influences brain structure and function measured by neuroimaging quantitative traits (QT). Given the high dimensionality of both imaging and genetic data, we propose to study Imaging Genetic Enrichment Analysis (IGEA), a new enrichment analysis paradigm that jointly considers meaningful gene sets (GS) and brain circuits (BC) and examines whether any given GS–BC pair is enriched in a list of gene–QT findings. Using gene expression data from Allen Human Brain Atlas and imaging genetics data from Alzheimer’s Disease Neuroimaging Initiative as test beds, we present an IGEA framework and conduct a proof-of-concept study. This empirical study identifies 25 significant high-level two-dimensional imaging genetics modules. Many of these modules are relevant to a variety of neurobiological pathways or neurodegenerative diseases, showing the promise of the proposal framework for providing insight into the mechanism of complex diseases.

## Introduction

Brain imaging genetics is an emerging field that studies how genetic variation influences brain structure and function. Genome-wide association studies (GWAS) have been performed to identify genetic markers such as single nucleotide polymorphisms (SNPs) that are associated with brain imaging quantitative traits (QTs) [20, 21]. Using biological pathways and networks as prior knowledge, enrichment analysis has also been performed to discover pathways or network modules enriched by GWAS findings to enhance statistical power and help biological interpretation [6]. For example, numerous studies on complex diseases have demonstrated that genes functioning in the same pathway can influence imaging QTs collectively even when constituent SNPs do not show significant association individually [18]. Enrichment analysis can also help identify relevant pathways and improve mechanistic understanding of underlying neurobiology [7, 11, 15, 19].

In the genetic domain, enrichment analysis has been widely studied in gene expression data analysis and has recently been modified to analyze GWAS data. GWAS-based enrichment analysis first maps SNP-level scores to gene-based scores, and then tests whether a pre-defined gene set *S* (e.g., a pathway) is enriched in a set of significant genes *L* (e.g., GWAS findings). Two strategies are often used to compute enrichment significance: threshold-based [4, 5, 9, 24] and rank-based [23]. Threshold-based approaches aim to solve an independence test problem (e.g., chi-square test, hypergeometric test, or binomial *z*-test) by treating genes as significant if their scores exceed a threshold. Rank-based methods take into account the score of each gene to determine if the members of *S* are randomly distributed throughout *L*.

In brain imaging genetics, the above enrichment analysis methods are applicable only to genetic findings associated with each single imaging QT. Our ultimate goal is to discover high-level associations between meaningful gene sets (GS) and brain circuits (BC), which typically include multiple genes and multiple QTs. To achieve this goal, we propose to study Imaging Genetic Enrichment Analysis (IGEA), a new enrichment analysis paradigm that jointly considers sets of interest (i.e., GS and BC) in both genetic and imaging domains and examines whether any given GS–BC pair is enriched in a list of gene–QT findings.

**Fig. 1:**
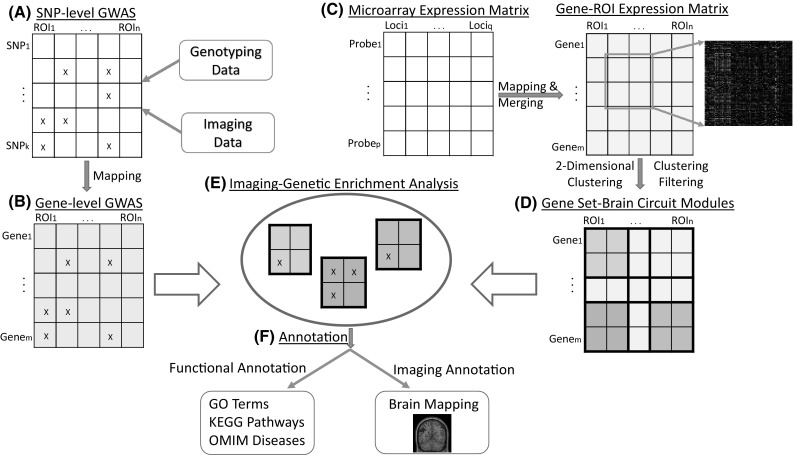
Overview of the proposed Imaging Genetic Enrichment Analysis (IGEA) framework. **A** Perform SNP-level GWAS of brain wide imaging measures. **B** Map SNP-level GWAS findings to gene-based. **C** Construct gene-ROI expression matrix from AHBA data. **D** Construct GS–BC modules by performing 2D hierarchical clustering, and then filter out 2D clusters with an average correlation below a user-given threshold. **E** Perform IGEA by mapping gene-based GWAS findings to the identified GS–BC modules. **F** For each enriched GS–BC module, examine the GS using GO terms, KEGG pathways, and OMIM disease databases, and map the BC to the brain

Using whole brain whole genome gene expression data from Allen Human Brain Atlas (AHBA) and imaging genetics data from Alzheimer’s Disease Neuroimaging Initiative (ADNI) as test beds, we present a novel IGEA framework and conduct a proof-of-concept study to explore high-level imaging genetic associations based on brain wide genome-wide association study (BWGWAS) results. For consistency purpose, in this paper, we use GS to indicate a set of genes and BC to indicate a set of regions of interest (ROIs) in the brain. The proposed framework consists of the following steps (see also Fig. 1): (1) conduct BWGWAS on ADNI amyloid imaging genetics data to identify SNP-QT and gene–QT associations, (2) use AHBA to identify meaningful GS–BC modules, (3) perform IGEA to identify GS–BC modules significantly enriched by gene–QT associations using a threshold-based strategy, and (4) visualize and interpret the identified GS–BC modules.

## Methods and materials

We write matrices and vectors as bold uppercase and lowercase letters, respectively. Given a matrix ${\mathbf {M}} = [m_{ij}]$, we denote its *i*th row as ${\mathbf {m}}^{i}$ and *j*th column as ${\mathbf {m}}_{j}$. Given two column vectors $\mathbf {a}$ and $\mathbf {b}$, we use ${\text {corr}}(\mathbf {a},\mathbf {b})$ to denote their Pearson’s correlation coefficient.

### Brain Wide Genome-Wide Association Study (BWGWAS)

The imaging and genotyping data used for BWGWAS were obtained from the Alzheimer’s Disease Neuroimaging Initiative (ADNI) database (adni.loni.usc.edu). The ADNI was launched in 2003 as a public–private partnership, led by Principal Investigator Michael W. Weiner, MD. The primary goal of ADNI has been to test whether serial magnetic resonance imaging (MRI), positron emission tomography (PET), other biological markers, and clinical and neuropsychological assessment can be combined to measure the progression of mild cognitive impairment (MCI) and early Alzheimer’s disease (AD). For up-to-date information, see http://www.adni-info.org.

Preprocessed [18F]Florbetapir PET scans (i.e., amyloid imaging data) were downloaded from adni.loni.usc.edu, then aligned to the corresponding MRI scans and normalized to the Montreal Neurological Institute (MNI) space as $2\times 2\times 2$ mm voxels. ROI level amyloid measurements were further extracted based on the MarsBaR AAL atlas. Genotype data of both ADNI-1 and ADNI-GO/2 phases were also downloaded, and then quality controlled, imputed, and combined as described in [10]. A total of 980 non-Hispanic Caucasian participants with both complete amyloid measurements and genome-wide data were studied. Associations between 105 (out of a total 116) baseline amyloid measures and 5,574,300 SNPs were examined by performing SNP-based GWAS using PLINK [17] with sex, age, and education as covariates. To facilitate the subsequent enrichment analysis, a gene-based *p* value was determined as the smallest *p* value of all SNPs located in $\pm 20$ K bp of the gene [14].

**Fig. 2:**
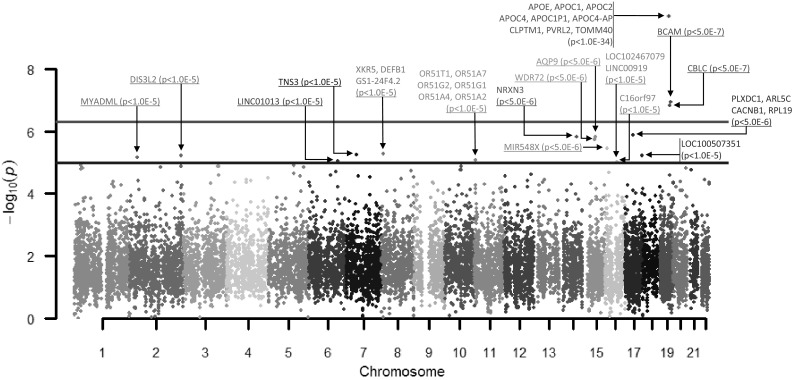
Manhattan plot of imaging quantitative genome-wide association for Alzheimer’s Disease individuals based on Precuneus (*right*) measurement from amyloid imaging data. The *x* axis represents the chromosomes and the *y* axis represents $-\log _{10}(P)$, where *P* is the gene-based significance

### Constructing GS–BC modules using AHBA

There are many types of prior knowledge that can be used to define meaningful GS and BC entities. In the genomic domain, the prior knowledge could be based on Gene Ontology or functional annotation databases; in the imaging domain, the prior knowledge could be neuroanatomic ontology or brain databases. In this work, to demonstrate the proposed IGEA framework, we use gene expression data from the Allen Human Brain Atlas (AHBA, Allen Institute for Brain Science, Seattle, WA, USA; available from http://www.brain-map.org/) to extract GS and BC modules such that genes within a GS share similar expression profiles and so do ROIs within a BC. We hypothesize that, given these similar co-expression patterns across genes and ROIs, each GS–BC pair forms an interesting high-level imaging genetic entity that may be related to certain biological function and can serve as a valuable candidate for two-dimensional IGEA.

The AHBA includes genome-wide microarray-based expression covering the entire brain through systematic sampling of regional tissue. Expression profiles for eight health human brains have been released, including two full brains and six right hemispheres. One goal of AHBA is to combine genomics with the neuroanatomy to better understand the connections between genes and brain functioning. As an early report indicated that individuals share as much as 95 % gene expression profile [28], in this study, we only included one full brain (H0351.2001) to construct GS–BC modules. First all the brain samples ($\sim$900) were mapped to MarsBaR AAL atlas, which included 116 brain ROIs. Due to many-to-one mapping from brain samples to AAL ROIs, there are >1 samples for each ROI. Following [27], samples located in the same ROI were merged using the mean statistics. Probes were then merged to genes using the same strategy. Finally, the preprocessed gene-ROI profiles were normalized for each ROI. As a result, the expression matrix contained 16,076 genes over 105 ROIs.

We use $\mathbf {E}$ to denote this expression matrix, where ${\mathbf {e}}^{i}$ is the expression level of gene *i* across all the 105 ROIs in $\mathbf {E}$, and ${\mathbf {e}}_{j}$ is the expression profile of ROI *j* across all the 16,076 genes in $\mathbf {E}$. Given two genes $i_1$ and $i_2$, we use the Pearson correlation coefficient to define their dissimilarity $d_{\text{ gene }}(i_1,i_2)$ as follows:

1 $$\begin{aligned} d_{\text{ gene }}(i_1,i_2) = 1/2\times (1 - \text{ corr }(({\mathbf {e}}^{i_1})^T,({\mathbf {e}}^{i_2})^T)). \end{aligned}$$

Similarly, given two ROIs $j_1$ and $j_2$, we define their dissimilarity $d_{\text{ roi }}(j_1,j_2)$ as follows:

2 $$\begin{aligned} d_{\text{ roi }}(j_1,j_2) = 1/2\times (1 - \text{ corr }({\mathbf {e}}_{j_1},{\mathbf {e}}_{j_2})). \end{aligned}$$

We performed a 2D cluster analysis on $\mathbf {E}$ to identify interesting GS–BC modules. First, we calculated the distance matrices for both genes and ROIs, using Eqs. (1) and (2), respectively. Next, two dendrograms were constructed by applying hierarchical clustering to two distance matrices separately, using the UPGMA (Unweighted Pair Group Method with Arithmetic Mean) algorithm [22]. After that, in the genomic domain, as most enrichment analyses placed constraints on genetic pathways of sizes from 10 to 200 [18], we cut the dendrogram at half of its height to build genetic clusters (i.e., GSs) whose sizes are mostly within the above range. Finally, in the imaging domain, we also employed the same parameter to construct ROI clusters (i.e., BCs).

Let $\mathbf {X}$ be a GS–BC module with *n* genes and *m* ROIs, where ${\mathbf {x}}^{i}$ is the expression level of gene *i* across all the *m* ROIs in $\mathbf {X}$, and ${\mathbf {x}}_{j}$ is the expression profile of ROI *j* across all the *n* genes in $\mathbf {X}$. For each pair of genes in $\mathbf {X}$, i.e., $(({\mathbf {x}}^{i_1})^T,({\mathbf {x}}^{i_2})^T)$, we calculate its correlation coefficient. For each pair of ROIs in $\mathbf {X}$, i.e., $({\mathbf {x}}_{j_1},{\mathbf {x}}_{j_2})$, we also calculate its correlation coefficient. After that, we transform each of these correlation coefficients, say *c*, to Fisher’s *z*-statistic *z*(*c*) using the following Eq. (3):

3 $$\begin{aligned} z(c) = \frac{1}{2} \log \left( \frac{1+c}{1-c}\right) . \end{aligned}$$

We then define $\overline{z}_{\text{ gene }}(\mathbf {X})$, the gene-based average Fisher’s *z*-statistics of correlation coefficient of $\mathbf {X}$, as follows:

4 $$\begin{aligned} \overline{z}_{\text{ gene }}({\mathbf {X}})=\frac{2}{n(n-1)}\sum _{0<i_1<i_2\le n} z(\text{corr }(({\mathbf {x}}^{i_1})^T({\mathbf {x}}^{i_2})^T)). \end{aligned}$$

Similarly, we define $\overline{z}_{\text{ roi }}({\mathbf {X}})$, the ROI-based average Fisher’s *z*-statistics of correlation coefficient of $\mathbf {X}$, as follows:

5 $$\begin{aligned} \overline{z}_{\text{ roi }}({\mathbf {X}})=\frac{2}{m(m-1)}\sum _{0<j_1<j_2\le m} z(\text{corr }({\mathbf {x}}_{j_1},\mathbf {x}_{j_2})). \end{aligned}$$

Based on these average gene-based and ROI-based *z*-statistics, respectively, we select the top 20 % of all the GS–BC modules and include those in our subsequent analyses, to ensure our studied modules have comparatively high co-expression profiles. Thus, in this work, we focus on the analysis of the following three types of GS–BC modules with top *z*-statistics:

1. *Gene-based* These are the modules with relatively high co-expression profiles between genes, i.e., $\overline{z}_{\text{ gene }}(\mathbf {X})$ is ranked in the top 20 % of all the $\overline{z}_{\text{ gene }}$ scores.
2. *ROI-based* These are the modules with relatively high co-expression profiles between ROIs, i.e., $\overline{z}_{\text{ roi }}(\mathbf {X})$ is ranked in the top 20 % of all the $\overline{z}_{\text{ roi }}$ scores.
3. *Gene and ROI-based* Both (1) and (2) hold.

**Fig. 3:**
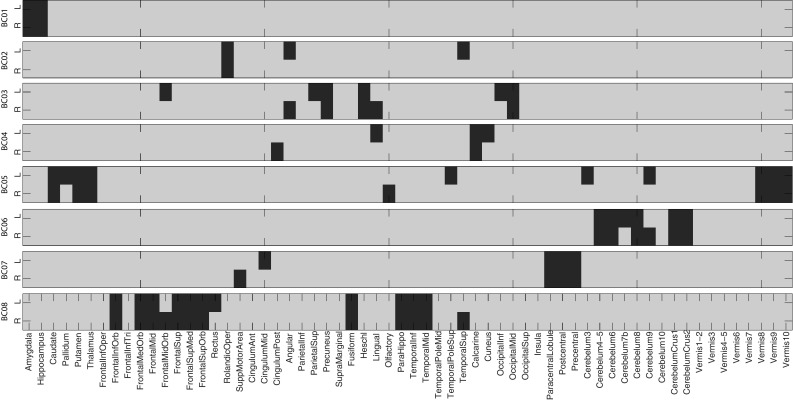
Eight unique brain circuits (BCs) identified from IGEA. ROIs belonging to each BC are colored in *red*

### Imaging Genetic Enrichment Analysis (IGEA)

Pathway enrichment analysis has been extensively employed to genomic domain to analyze the genetic findings associated with a specific imaging QT. In this study, our goal is to identify high-level associations between gene sets and brain circuits, which typically include multiple genes and multiple QTs.

In this study, we propose the threshold-based IGEA by extending the existing threshold-based enrichment analysis. SNP-level findings have been mapped to gene level findings in Sect. 2.1. The GWAS findings are a list *L* of $N=N_G \times N_B$ gene–QT associations, where we have a set $G_d$ of $N_G=|G_d|$ genes and a set $B_d$ of $N_B=|B_d|$ QTs in our analysis. From Sect. 2.2, GS–BC modules have been constructed, where either relevant genes share similar expression profiles across relevant ROIs, or relevant ROIs share similar expression profiles across relevant genes, or both. Given an interesting GS–BC module with gene set $G_{k}$ and QT set $B_{k}$, IGEA aims to determine whether the target GS–BC module $T=\{(g,b)|g\in G_d \cap G_k, b \in B_d \cap B_k\}$ is enriched in *L*.

Now we describe our threshold-based IGEA method. We have *N* gene–QT pairs from GWAS. Out of these, $n=|A|$ pairs (the set *A*) are significant ones with GWAS *p* value passed a certain threshold. We also have $m=|P|$ (the set *P*) gene–QT pairs from a given GS–BC module, and *k* significant pairs are from *P*. Using Fisher’s exact test for independence, the enrichment *p* value for the given GS–BC module is calculated as follows:

6 $$\begin{aligned} {p{\text{-value}}}={\mathrm{Pr}}(|A \cap P|\ge k) =\sum \nolimits _{i\ge k} \frac{{m\atopwithdelims ()i} \times {N-m \atopwithdelims ()n-i}}{{N\atopwithdelims ()n}}. \end{aligned}$$

Here, we use Pr$(\cdot )$ to denote the probability function.

### Evaluation of the identified GS–BC modules

For evaluation purpose, we tested the statistical significance of the IGEA results. We hypothesize that the gene–QT associations from BWGWAS of the original data should be overrepresented in certain GS–BC modules, and the BWGWAS results on permuted data should not be enriched in a similar number of GS–BC modules. We performed the IGEA analysis on $n=50$ permuted BWGWAS results, and estimated the *p* value for the number of significant GS–BC modules discovered from the original data using a *t*-distribution with $n-1$ degrees of freedom.

Given a BWGWAS result *R*, let Prop(*R*) be the proportion of modules which are significantly enriched by *R*. Let $R_{\text{ orig }}$ be the original BWGWAS result, and $R_{\text{ perm }(i)}$ be the *i*th permuted BWGWAS result. Let $S = \{ {\mathrm{Prop}}(R_{\text{ perm }(i)}) \mid 1 \le i \le n\}$ be the set of these proportion values for all the permuted results. Then the *p* value is estimated using Eq. (7).

7 $$\begin{aligned} p\text{-value } = {\mathrm{Pr}}\left( T_{n-1} \ge \frac{{\mathrm{Prop}}(R_{\text{ orig }})-\mu _{\text{ perm }}}{\sqrt{1+1/n}\times \sigma _{\text{ perm }}}\right) . \end{aligned}$$

where $T_{n-1}$ is the *t*-distribution with $n-1$ degrees of freedom, $\mu _{\text{ perm }}$ is the sample mean of *S*, and $\sigma _{\text{ perm }}$ is the sample standard deviation of *S*.

To determine the functional relevance of the enriched GS–BC modules, we also tested whether genes from each module are overrepresented for specific neurobiological functions, signaling pathways, or complex neurodegenerative diseases. We performed pathway enrichment tests using gene ontology (GO) terms, KEGG pathways and OMIM (Online Mend-elian Inheritance in Man) database.

**Table 1:** Twenty-five significantly enriched GS–BC modules from IGEA

Module ID	Top 20 % Co-expressed^a^	BC ID	# of ROIs	GS ID	# of genes	Corrected *P* value (gene-based)	Corrected *P* value (ROI-based)	Corrected *P* value (Gene&ROI-based)
01	R^c^	BC07	8	GS01	81	–	2.61E$-$06	–
02	G, R, G&R^d^	BC02	4	GS02	168	9.06E$-$06	9.06E$-$06	9.06E$-$06
03	G^b^	BC03	11	GS02	168	2.54E$-$11	–	–
04	G, R, G&R	BC04	5	GS02	168	1.44E$-$06	1.44E$-$06	1.44E$-$06
05	G	BC05	14	GS02	168	6.42E$-$06	–	–
06	R	BC06	13	GS02	168	–	5.91E$-$07	–
07	R	BC08	23	GS02	168	–	5.65E$-$22	–
08	G, R, G&R	BC01	4	GS03	55	1.38E$-$06	1.38E$-$06	1.38E$-$06
09	G	BC02	4	GS03	55	4.39E$-$13	–	–
10	R	BC04	5	GS03	55	–	1.41E$-$15	–
11	G	BC05	14	GS03	55	1.01E$-$14	–	–
12	R	BC06	13	GS03	55	–	1.72E$-$08	–
13	R	BC07	8	GS03	55	–	2.40E$-$21	–
14	R	BC07	8	GS04	66	–	4.00E$-$07	–
15	G, R, G&R	BC01	4	GS05	19	3.83E$-$05	3.83E$-$05	3.83E$-$05
16	G, R, G&R	BC02	4	GS05	19	6.88E$-$09	6.88E$-$09	6.88E$-$09
17	G, R, G&R	BC04	5	GS05	19	2.64E$-$10	2.64E$-$10	2.64E$-$10
18	R	BC06	13	GS05	19	–	2.26E$-$11	–
19	G, R, G&R	BC07	8	GS05	19	1.54E$-$14	1.54E$-$14	1.54E$-$14
20	G, R, G&R	BC02	4	GS06	28	4.87E$-$08	4.87E$-$08	4.87E$-$08
21	G	BC02	4	GS07	24	7.69E$-$05	–	–
22	G&R	BC01	4	GS08	33	–	–	1.97E$-$04
23	G	BC02	4	GS08	33	1.11E$-$07	–	–
24	R	BC04	5	GS08	33	–	7.39E$-$09	–
25	G	BC02	4	GS09	111	4.07E$-$05	–	–

See also Sect. 3.2 and Fig. 3 for details about relevant GSs and BCs, respectively

^a^To indicate whether the top 20 % modules are selected based on the gene-based, ROI-based, or gene&ROI-based strategy

^b^G: Gene-based

^c^R: ROI-based

^d^G&R: Gene&ROI-based

**Fig. 4:**
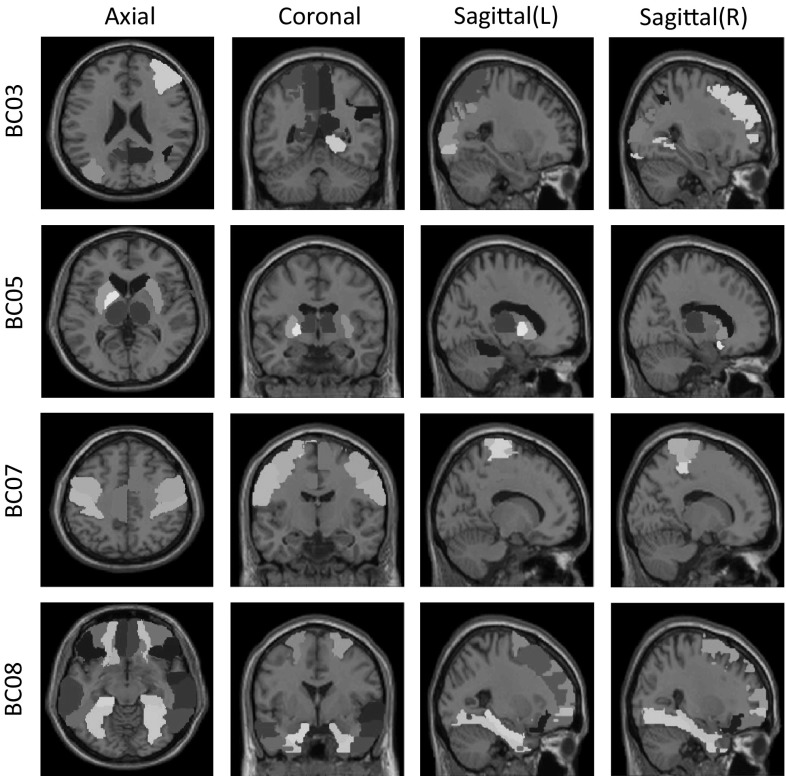
Brain maps of four brain circuits (BCs) identified from IGEA

## Results

### Significant GS–BC modules

By performing hierarchical clustering on both genetic and imaging domains, 171 out of 216 genetic clusters (only those with size ranging from 10 to 200) and 9 imaging clusters (with size ranging from 4 to 23, no clusters are excluded) were identified. 1539 GS–BC modules were generated by combining each pair of genetic and ROI clusters. Two sets of 308 (20 % of 1539) modules were selected according to gene-based and ROI-based z-statistics, respectively. Among them, 90 modules were among top 20 % in both gene-based and ROI-based ranking results. We used a moderate size threshold for the selection, to avoid the exclusion of potentially interesting candidates.

For the BWGWAS results, we obtained $16,076 \times 105=1,687,980$ gene–QT associations after mapping SNP-based *p* values to genes. Out of these, 1402 gene–QT associations passed the BWGWAS *p* value of ${1.0}{\text{e-}}{5}$. Figure 2 shows the gene-based GWAS result of an example QT (i.e., the average amyloid deposition in the right precuneus). Precuneus amyloid concentration has been demonstrated to be associated with disordered activity in Alzheimer’s Disease [8].

Three sets of constructed GS–BC modules (308, 308, and 90 with top z-statistics using gene-based, ROI-based, and gene&ROI-based strategies, respectively, see Sect. 2.2) were tested separately for whether they could be enriched by BWGWAS results using IGEA. Across three sets, totally 25 modules turned out to be significant after Bonferroni correction (see Table 1), of which 15, 17, and 9 are from gene-based, ROI-based, and both gene&ROI-based categories, respectively. We also tested the significance of the number of identified GS–BC modules. Compared to the permuted BWGWAS results, the analysis on the original data yielded a significantly larger number of enriched GS–BC modules with estimated *p* values of $7.6\text{e-}25, 1.2\text{e-}9$, and $1.8\text{e-}25$, corresponding to gene-based, ROI-based, and gene&ROI-based strategies, respectively, indicating that imaging genetic associations existed in these enriched GS–BC modules.

**Table 2:** Top enriched OMIM diseases of identified GSs

GS ID	# of gene	OMIM Disease	*P* value
GS01	81	Encephalopathy	4.2E$-$2*
		Dementia	3.6E$-$2*
GS02	168	Encephalopathy	5.0E$-$2
		Breast cancer	9.5E$-$2
GS03	55	Leukemia	2.7E$-$2*
		Alzheimer’s disease	8.9E$-$2
GS04	66	Hypertension	5.0E$-$2
GS05	19	Anomalies	2.4E$-$2*
		Alzheimer’s disease	4.5E$-$2*
GS06	28	Ectodermal dysplasia	2.0E$-$2*
GS07	24	Hypertension	3.4E$-$2*
		Spinocerebellar ataxia	4.3E$-$2^*^
GS08	33	Glycogen storage disease	1.6E$-$2*
GS09	111	Immunodeficiency	1.4E$-$2*

* Significantly enriched

Across all 25 identified modules, there are 9 and 8 unique GS and BC entities, respectively. Figure 3 shows the 8 unique identified BCs with corresponding ROI names, and Fig. 4 maps four of those onto the brain. For example, BC03 and BC04 include structures that are major spots for amyloid accumulation in AD (e.g., cingulum, precuneus). BC05 involves structures responsible for motivated behaviors (e.g., caudate, pallidum, putamen) and sensory information processing (e.g., thalamus). BC08 involves various frontal regions responsible for executive functions. Details of all 25 modules are listed in Table 1. We can find that some modules share common gene sets with different brain circuits, and some share the same brain circuits with different gene sets. This illustrates the complex associations among multiple genes and multiple brain ROIs.

**Fig. 5:**
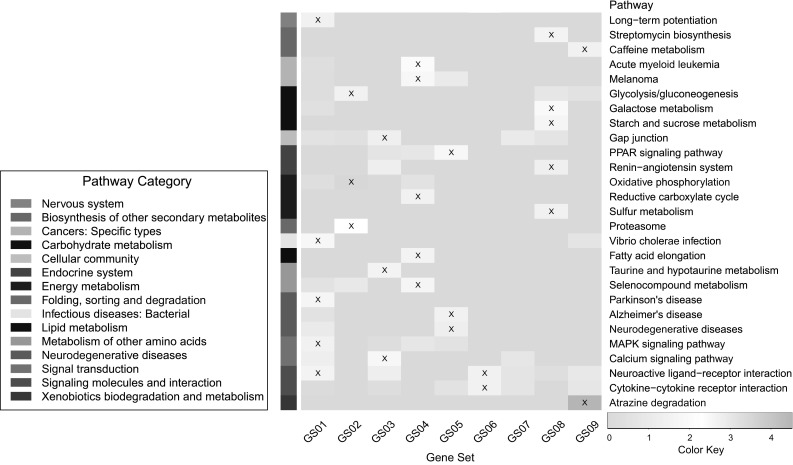
Results of KEGG pathway enrichment for identified GSs. The *x* axis represents unique GS ID, and *y* axis represents −log *p* value of enrichment significance of KEGG pathways. *Marked cell* represents significant enrichment (*p* value $<0.05$)

### Pathway analysis of identified GS–BC modules

To explore and analyze functional relevance of our identified GS–BC modules, we performed pathway enrichment analysis from three aspects including GO terms, functional pathways and diseases using Gene Ontology (GO), KEGG pathways, and OMIM diseases databases, respectively.

Figure 5 shows the KEGG pathway enrichment results which were mapped to 15 categories. From the results, most identified GSs had a number of significant functional enrichments. Several of them were directly related to the neurodegenerative disease and its development, e.g., Alzheimer’s Disease enriched in GS05 and Parkinson’s Disease enriched in GS01. Another major part of them were also related to the neurodegenerative diseases and their development. For instance, caffeine as the most widely used psychoactive substance, its metabolism (from GS09 located in Module 25) can affect brain metabolism and has potential benefits on Parkinson’s Disease treatment [16]. There are also several enriched pathways related to oxidative stress, which is a critical factor for a range of neurodegenerative disorders. For example, glycolysis and gluconeogenesis (from GS02 located in Modules 02-07) are associated with hypoxia, ischemia, and AD [2]. Gap junctions (from GS03 located in Modules 08-13) can couple various kinds of cells in the central nervous system (CNS) which play an important role in maintaining normal function. Signaling transduction, like calcium signaling pathway (from GS03 located in Modules 08-13) playing key role in short- and long-term synaptic plasticity has shown abnormality in many neurodegenerative disorders including Alzheimer’s Disease, Parkinson’s disease, amyotrophic lateral sclerosis (ALS), Huntington’s disease, spinocerebellar ataxias (SCA), and so on [1].

Table 2 shows the OMIM disease enrichment results. Several neurodegeneration-related and age-related diseases and complex disorders were enriched in various gene sets, such as Alzheimer’s Diease from GS03 and GS05, Encephalopathy from GS01 and GS02, and Anomalies from GS05. Besides neurodegeneration diseases and disorders, several cancer-related entities are detected including breast cancer from GS02 and leukemia from GS03. These findings provided potential evidence for the studies that focused on investigating the relationship between cancer and neurodegeneration, with abnormal cell growth and cell loss in common.

**Table 3:** Top enriched GO terms of GSs from identified GS–BC modules

Group	GS ID	# of genes	GO Category	Corrected *p* value
Behavior	GS03	55	Behavior	2.2E$-$2
			Learning or memory	4.4E$-$2
Cell communication	GS01	81	Regulation of synaptic transmission	2.7E$-$6
			Neuron-neuron Synaptic transmission	2.9E$-$3
	GS03	55	Synaptic transmission	1.7E$-$4
Metabolic process	GS05	19	Fat-soluble vitamin metabolic process	4.3E$-$2
			Organic hydroxy compound biosynthetic process	4.8E$-$2
	GS06	28	Regulation of translational termination	2.8E$-$2
Mitochondrion	GS02	168	Mitochondrial membrane part	2.5E$-$3
			Mitochondrial respiratory chain complex I	4.9E$-$3
Neurological system process	GS03	55	Associative learning	1.1E$-$2
			Learning	4.5E$-$6
	GS09	111	Detection of chemical stimulus involved in sensory perception	1.1E$-$4
			Olfactory receptor activity	1.9E$-$5
Response to stimulus	GS03	55	Response to amphetamine	2.0E$-$3
			Visual behavior	4.5E$-$3
	GS05	19	Response to cholesterol	3.6E$-$2
			Response to sterol	3.7E$-$2
	GS09	111	Detection of chemical stimulus	1.6E$-$4
Signal transduction	GS01	81	Glutamate receptor signaling pathway	7.3E$-$4
	GS03	55	Adenylate cyclase-activating dopamine receptor signaling pathway	3.1E$-$3
			Dopamine receptor signaling pathway	1.4E$-$2
	GS05	19	Transmembrane receptor protein kinase activity	4.4E$-$2
	GS09	111	Olfactory receptor activity	1.9E$-$5

Gene Ontology (GO) enrichment indicates the relationship between identified gene sets and GO terms from three categories including biological process (BP), cellular component (CC), and molecular function (MF) (http://geneontology.org/). For the GO enrichment of all 9 gene sets, 163 various GO terms were significantly enriched. Top enriched terms were selected and grouped to 7 categories including behavior, cell communication, mitochondrion, metabolic process, neurological system process, response to stimulus, and signal transduction, as shown in Table 3. A large number of these terms have direct or indirect relationships with neurodegenerative diseases or phenotypes.

## Discussion

We have presented a two-dimensional imaging genetic enrichment analysis (IGEA) framework to explore the high-level imaging genetic associations by integrating whole brain genomic, transcriptomic, and neuroanatomic data. Traditional pathway enrichment analysis focused on investigating genetic findings of a single phenotype one at a time, and relationships among imaging QTs could be ignored. Such approach could be inadequate to provide insights into the mechanisms of complex diseases that involve multiple genes and multiple QTs. In this paper, we have proposed a novel enrichment analysis paradigm IGEA to detect high-level associations between gene sets and brain circuits. By jointly considering the complex relationships between interlinked genetic markers and correlated brain imaging phenotypes, IGEA provides additional power for extracting biological insights on neurogenomic associations at a systems biology level. For example, let us take a look at GS03-BC05, an identified module significantly enriched by our GWAS findings. Several ROIs (e.g., caudate, pallidum, and putamen) from BC05 have been indicated responsible for motivated behaviors [3]. Meanwhile both KEGG and GO functional enrichment results of GS03 show high relevance to behavior and normal function maintaining (see Fig. 5; Table 3). These observations suggest that this high-level imaging genetic pattern could be relevant to the behavior mechanism. It warrants further investigation to perform analyses targeted at these genes and ROIs in independent cohorts to better understand the underlying mechanism from the imaging genetic perspective.

The real power of IGEA, however, can be affected by several aspects. First, the constructed GS–BC modules should reflect the real relationships among genes as well as brain ROIs. Thus, it is crucial to define meaningful gene sets and brain circuits. In our paper, GSs and BCs were separately extracted from AHBA brain wide expression data based on hierarchical clustering, which were then combined to provide GS–BC modules. This strategy was based on the idea that interlinked genetic markers (or brain ROIs) would conserve similar expression pattern, i.e., would be highly co-expressed. Second, the statistical measure of enrichment evaluation can be based on different strategies. We adopted hypergeometric test in our experiment to estimate the over-representation of our defined GS–BC modules to the list of gene–QT pair.

Based on these two considerations, our proposed paradigm can be further improved. From our GS–BC module construction, GSs (or BCs) are clustered together based on their co-expression pattern across all the ROIs in the whole brain (or across all the genes in the genome). Although statistical measures were calculated using Fisher’s z-transformation to restrict our analyses on only highly co-expressed modules from our bi-clustering results, we could be missing other highly co-expressed GSs (or BCs) if they only had similar expression patterns on a small set of ROIs (or genes). In other words, our module construction strategy considered the global expression pattern but ignored the local ones. It is worth for further investigation to try other reasonable strategies by applying prior knowledge such as pre-defined genetic pathways/networks or brain circuits, or by using different co-clustering algorithms (e.g., [26]) to take into consideration of relevant local expression patterns.

Hypergeometric test requires a pre-defined threshold to determine the list of gene–QT pairs. Another limitation is that it considers only the count of significant gene–QT pairs, but ignores the strength of gene–QT associations. There are a number of rank-based enrichment analysis methods (e.g., GSEA [23]) that can be employed in our two-dimensional enrichment analysis to overcome these disadvantages. Another issue is that we used the smallest SNP-level *p* value within the gene to represent the gene-based *p* value. Therefore, another possible future direction is to explore other set-based methods for calculating gene-based *p* values such as VEGAS [13], GATES [12], and so on. Besides, from mathematical perspective, associating GS–BC modules and gene–QT findings can be seen as a similarity discovery over two matrices. Thus, another future direction could be to study this problem using machine learning approaches similar to that proposed by Wang et al. [25].
